# MIA PaCa-2 and PANC-1 – pancreas ductal adenocarcinoma cell lines with neuroendocrine differentiation and somatostatin receptors

**DOI:** 10.1038/srep21648

**Published:** 2016-02-17

**Authors:** Rui Gradiz, Henriqueta C. Silva, Lina Carvalho, Maria Filomena Botelho, Anabela Mota-Pinto

**Affiliations:** 1 General Pathology Laboratory, Faculty of Medicine, University of Coimbra, Coimbra, Portugal; 2 Medical Genetics’ Unit, Faculty of Medicine, University of Coimbra, Coimbra, Portugal; 3 Anatomical and Molecular Pathology Department, Faculty of Medicine, University of Coimbra, Coimbra, Portugal; 4 Biophysics’ Unit, Faculty of Medicine, University of Coimbra, Coimbra, Portugal; 5 CIMAGO – Research Center for Environment, Genetics and Oncobiology, Faculty of Medicine, University of Coimbra, Coimbra, Portugal; 6 CNC.IBILI, University of Coimbra, Portugal

## Abstract

Studies using cell lines should always characterize these cells to ensure that the results are not distorted by unexpected morphological or genetic changes possibly due to culture time or passage number. Thus, the aim of this study was to describe those MIA PaCa-2 and PANC-1 cell line phenotype and genotype characteristics that may play a crucial role in pancreatic cancer therapeutic assays, namely neuroendocrine chemotherapy and peptide receptor radionuclide therapy. Epithelial, mesenchymal, endocrine and stem cell marker characterization was performed by immunohistochemistry and flow cytometry, and genotyping by PCR, gene sequencing and capillary electrophoresis. MIA PaCa-2 (polymorphism) expresses CK5.6, AE1/AE3, E-cadherin, vimentin, chromogranin A, synaptophysin, SSTR2 and NTR1 but not CD56. PANC-1 (pleomorphism) expresses CK5.6, MNF-116, vimentin, chromogranin A, CD56 and SSTR2 but not E-cadherin, synaptophysin or NTR1. MIA PaCA-1 is CD24^−^, CD44^+/++^, CD326^−/+^ and CD133/1^−^, while PANC-1 is CD24^−/+^, CD44^+^, CD326^−/+^ and CD133/1^−^. Both cell lines have *KRAS* and *TP53* mutations and homozygous deletions including the first 3 exons of *CDKN2A/p16*^*INK4A*^, but no *SMAD4/DPC4* mutations or microsatellite instability. Both have neuroendocrine differentiation and SSTR2 receptors, precisely the features making them suitable for the therapies we propose to assay in future studies.

Pancreatic ductal adenocarcinoma accounts for over 90% of all pancreatic cancers^1^ and is now the fourth leading cause of cancer-related death in the western world^2^,^3^. The prognosis is extremely poor, with a 5-year relative survival rate of 5%^2^ and median survival of 3.5 months for non-resectable tumors^4^. Surgical resection is the only potentially curative therapy^5^, but even in these cases relapses are common^6^.

Pancreatic ductal adenocarcinomas can display disseminated neuroendocrine cells. However, the relative incidence, histogenesis, hormone production and prognostic implications of their presence are controversial^7^. Antibodies against the neural cell adhesion molecule (NCAM or CD56), neuronspecific enolase (NSE), synaptophysin, CD57 and chromogranin A (CGA) can be used for immunostaining and identification of neuroendocrine cells in pancreatic ductal adenocarcinoma^7^,^8^.

Although the putative cell of origin for pancreatic cancer remains elusive, within hematopoietic and solid tumors *minor* subpopulations of cells with a self-renewing capacity, also termed cancer stem cells (CSCs), have been identified, which appear to be responsible for tumor initiation, growth, metastases and resistance to conventional therapies^9^. Different subpopulations of cancer stem cells have been identified in pancreatic ductal adenocarcinoma, based on the use of a combination of surface markers, such as CD44, CD24, EpCAM (Epithelial Cell Adhesion Molecule), also known as ESA (Epithelial Cell Surface Antigen), CD326, CD133 and others, such as side population (SP) cells with overexpression of ABCG2 transporters and CXCR4. These markers allow CSC isolation, propagation and characterization^10^ to be carried out.

CSCs may be dependent on the phenotype for epithelial-mesenchymal transition (EMT), a primordial developmental process by which adult polarized epithelial cells undergo biochemical changes and assume a mesenchymal phenotype, acquiring an increased migratory capacity, invasiveness, resistance to apoptosis and expression of extracellular matrix components^11^. This dedifferentiation process is associated with a loss of functional epithelial cell markers, such as E-cadherin, and increased expression of mesenchymal markers, such as vimentin^12^. EMT has also been demonstrated to correlate with CD24^+^CD44^+^ and CD133^+^ cells in pancreatic cancer, providing pancreatic cancer stem cells with a strong migratory capacity, while maintaining their ability to multiply and thus allowing the production of progenies during metastasis^13^.

Within pancreatic cells, the accumulation of genetic changes leads to a multi-staged process giving rise to malignancy. Mutations in the proto-oncogene *KRAS* and tumor suppressors *CDKN2A*, *TP53* and *SMAD4/DPC4* are major genetic alterations associated with cell cycle deregulation, apoptosis inhibition, invasion, metastasis and poor treatment response^14^. Microsatellite instability (MSI) is a genetic feature of sporadic and familial cancers of multiple sites and is related to defective mismatch repair (MMR) protein function^15^. It has been described in pancreatic cancer and studies suggest that MSI may be associated with *KRAS* mutations^16^. Other investigations propose that MSI-positive pancreatic cancers may have a better prognosis after resection because of the intense immunoreaction to the tumor^17^. Furthermore, the risk of developing a pancreatic tumor is 8.6 times more in patients with Lynch syndrome compared to the general population^18^. In these patients, a c.2252_2253delAA *MLH1* mutation associated with an increased risk of pancreatic tumors^19^ was found, and another in the *MSH2* gene associated with an intraductal papillary mucinous neoplasm^20^ was also discovered.

The existence of adenocarcinomas with neuroendocrine differentiation (NED) may provide the possibility of treating this subgroup of tumors with peptide receptor radionuclide therapy, alone or associated with other forms of treatment, such as chemotherapy^21^. This option of treatment requires the presence of high affinity receptors on tumoral cells, such as somatostatin receptors (SSTRs)^22^. Five different subclasses of these receptors, SSTR1 to 5, have been cloned and characterized, but in the majority of neuroendocrine gastroenteropancreatic tumors, SSTR2 and SSTR5 predominate^23^. The synthesis of somatostatin analogs, such as octreotide, has led to them being used in therapy in radiolabeled form^24^. ^90^Y [^90^Y-DOTA (tetraazacyclododecane-1,4,7,10-tetracetic acid)–TOC (Tyr^3^-octreotide)], a pure β emitter, and ^177^Lu [^177^Lu-DOTA-TATE (Tyr^3^-octreotate)], a β and γ emitter, are currently the most widely used radionuclides in the treatment with somatostatin analogs^25^.

Neurotensin receptors (NTRs) are present in the large majority of pancreatic ductal adenocarcinomas, but absent in endocrine pancreatic cancers, chronic pancreatitis and normal pancreatic acini^26^. Neurotensin is a 13-amino-acid peptide found in both the central nervous system and peripheral tissues, mainly in the gastrointestinal tract. At least three subtypes of NTRs have been cloned: NTR1, NTR2 and sortilin/NTR3^27^. One clinical evaluation study using a radiolabeled NT analog (^99^ ^m^Tc-NT-XI) included four exocrine pancreatic carcinoma patients, with moderate tumor uptake being observed in only one patient^28^. More recently, NTR1 was assumed to be a potential target for pharmacological therapy^29^, and in another study an ^18^F-labeled diarylpyrazole glycoconjugate obtained excellent stability and highly beneficial biodistribution *in vivo*, as demonstrated by PET imaging in HT-29 tumor bearing nude mice, representing a highly promising candidate for PET imaging of NTR1 positive tumors^30^.

MIA PaCa-2 with functional SSTR2 receptors^31^ and PANC-1 cell lines are primary tumors currently used as *in vitro* models to study pancreatic ductal adenocarcinoma carcinogenesis. Their morphological and genetic characteristics are well studied. However, studies using cell lines should always characterize these cells to ensure that the results are not distorted by unexpected morphological or genetic changes possibly due to culture time or passage number. Thus, the aim of this study was to confirm and investigate MIA PaCa-2 and PANC-1 cell line phenotype and genotype characteristics that may be relevant for their use as *in vitro* models in pancreatic cancer neuroendocrine chemotherapy and peptide receptor radionuclide therapy assays.

## Results

### Immunohistochemistry

**Figure 1:**
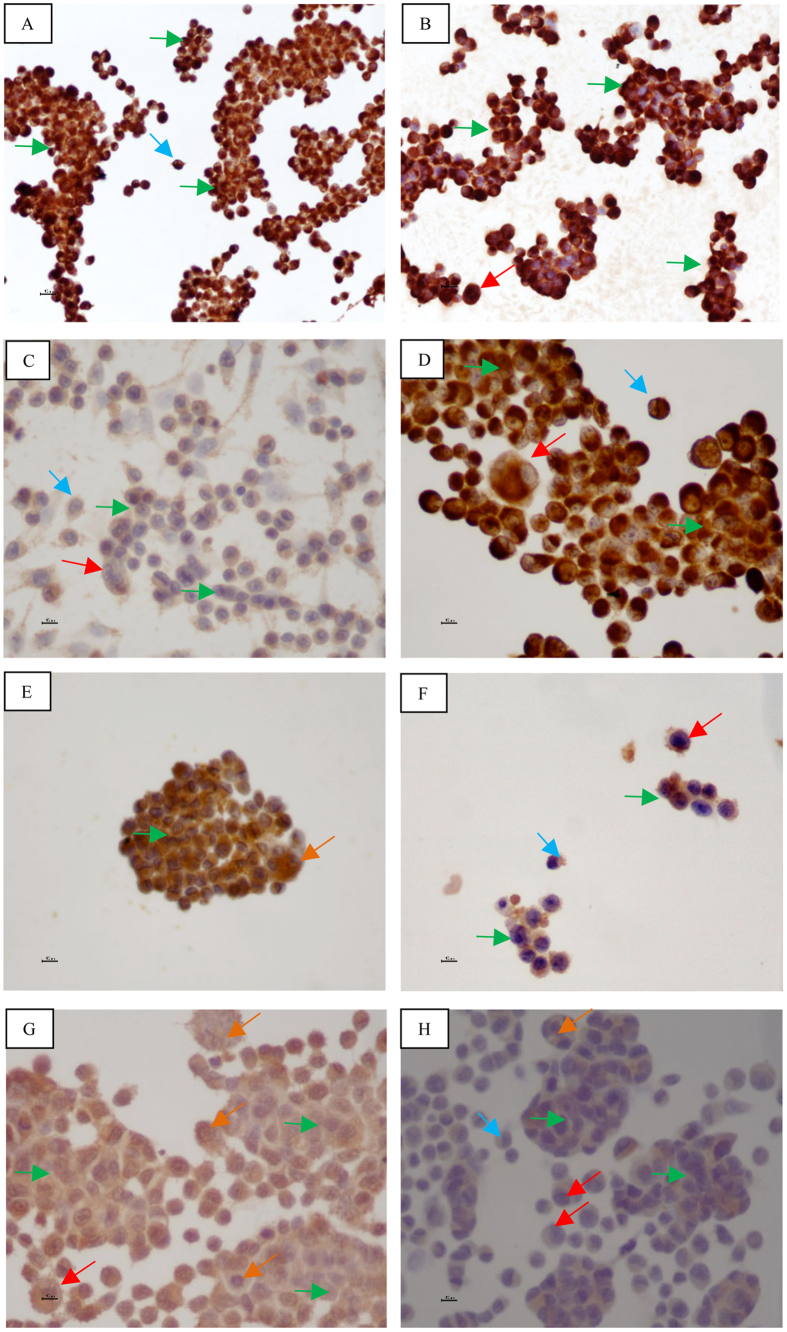
MIA PaCa-2: Immunohistochemistry. (**A**) CK 5.6 ×200; (**B**) AE1/AE3 ×200; (**C**) E-cadherin ×400; (**D**) Vimentin ×400; (**E**) Chromogranin A ×400; (**F**) Synaptophysin ×400; (**G**) SSTR2 ×400; (**H**) NTR1 ×400. Small single cells (blue arrow); small clustered cells (green arrow); large single cells (red arrow); large clustered cells (orange arrow). Scale bar: 10 μm.

Microscopic observation of MIA PaCa-2 cells revealed the presence of two distinct morphological patterns (Fig. 1): large cells and small cells (morula). The MIA PaCa-2 cell line expresses (Table 1): CK5.6 (Fig. 1A), AE1/AE3 (Fig. 1B), E-cadherin (Fig. 1C), vimentin (Fig. 1D), chromogranin A (Fig. 1E) and synaptophysin (Fig. 1F) but not CD56. It also expresses SSTR2 (Fig. 1G) and NTR1 (Fig. 1H).

**Figure 2:**
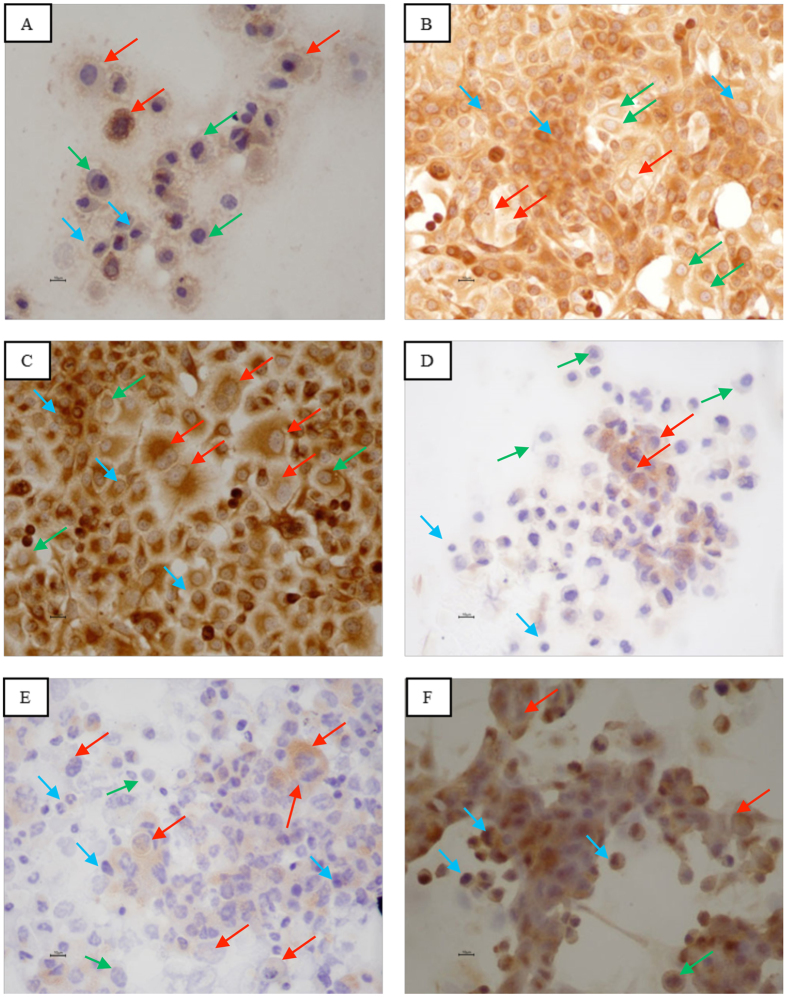
PANC-1: Immunohistochemistry. (**A**) CK 5.6 ×400; (**B**) AE1/AE3 ×200; (**C**) Vimentin ×200; (**D**) Chromogranin A ×400; (**E**) CD56 ×400; (**F**) SSTR2 ×400. Small cells (blue arrow); intermediate cells (green arrow); large cells (red arrow). Scale bar: 10 μm.

Microscopic observation of PANC-1 cells revealed the presence of three distinct morphological patterns (Fig. 2): large cells (isolated), intermediate cells (stellate) and small cells (morula). The PANC-1 cell line expresses (Table 2): CK5.6 (Fig. 2A), AE1/AE3 (Fig. 2B), vimentin (Fig. 2C), chromogranin A (Fig. 2D), CD56 (Fig. 2E) and SSTR2 (Fig. 2F) but not E-cadherin, synaptophysin or NTR1.

### Flow cytometry

Immunophenotyping of MIA PaCa-2 and PANC-1 cell lines revealed differences in morphology and immunohistochemistry. Flow cytometry was employed to clarify whether, in morphological terms, there were in fact two cell populations in MIA Paca-2 and three in PANC-1. This technique was also used to characterize these cell lines in terms of the presence of pancreatic stem cell markers, such as CD24, CD44, CD133/1 and CD326.

**Figure 3:**
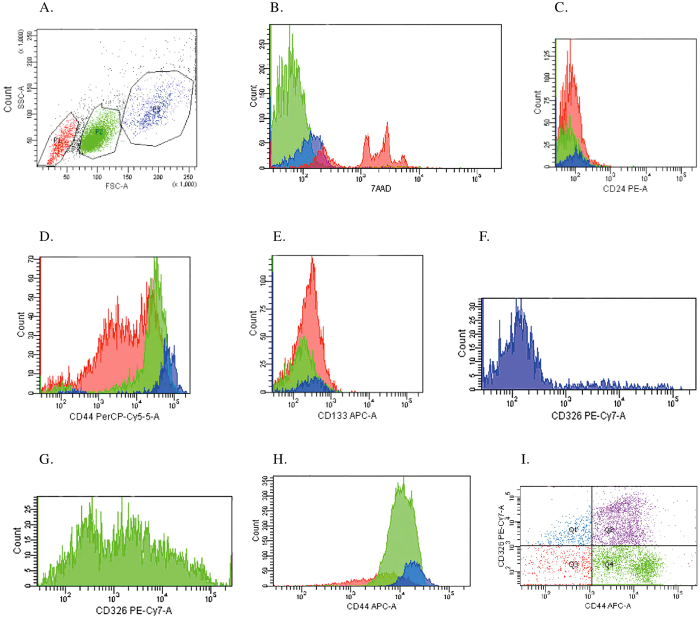
Flow cytometry analysis of morphology and stem cell marker expression on MIA PaCa-2 tumor cells. (**A**) Dot plot FSC/SSC representative of the populations P1 (red), P2 (green) and P3 (blue); (**B**) Histogram representative of 7AAD expression; (**C**) Histogram representative of CD24 expression; (**D**) Histogram representative of CD44 expression (fluorochrome PerCP/Cy5.5); (**E**) Histogram representative of CD133 expression; (**F**) Histogram representative of CD326 expression in P3; (**G**) Histogram representative of CD326 expression in P2; (**H**) Histogram representative of CD44 expression (fluorochrome APC); (**I**) CD44/CD326 expression.

The MIA PaCa-2 results (Fig. 3) demonstrated the presence of three groups of gated cells based on FSC *vs.* SSC characteristics (Fig. 3A): red (P1), green (P2) and blue (P3) populations, supporting the possibility of the existence of a polymorphic cell line. We obtained a total of 20,000 events, with P1 representing 2,539 events, corresponding to 12.7% of cells, with a Mean Fluorescence Intensity (MFI) of 5,559; P2, 12,868 events, 64.3% of cells, with an MFI of 12,158; and P3, 2,322 events, 11.6% of cells, with an MFI of 20,031. The smaller and less complex P1 (red) population stained positive with cell viability staining solution 7AAD (Fig. 3B), confirming that this group was composed of non-viable cells, while the population of small cells (green, P2) and large cells (blue, P3) stained negative for 7AAD, demonstrating that P2 and P3 are composed of viable cells.

To investigate the presence of stem cells, further flow cytometry studies were then conducted using specific MIA PaCa-2 stem cell markers such as CD24 PE (Fig. 3C), CD44 PerCP/Cy5.5 (Fig. 3D), CD133/1 APC (Fig. 3E) and CD326 PE-Cy7 (Fig. 3F,G). The results showed a lack of expression for CD24 (CD24^−^) (Fig. 3C) and CD133/1 (CD133/1^−^) (Fig. 3D). The large cell (blue) population showed higher levels of CD44 (Fig. 3D), represented as a CD44^++^ phenotype, and higher MFI (70.9) than the small cell (green) population, which showed a CD44^+^ phenotype and lower MFI (32.9) (Fig. 3D). The experiment was repeated with another fluorochrome (APC) (Fig. 3H) and the result was the same. In addition, concerning CD326 expression, it can be seen in Fig. 3F that the large cell population is mainly CD326^−^ and in Fig. 3G the small cell population is CD326^−/+^. Thus, it seems that the large cell population has the phenotype CD24^−^CD44^++^CD326^−^CD133/1^−^ and the small cell population, CD24^−^CD44^+^CD326^−/+^CD133/1^−^, making the subpopulations of the MIA PaCa-2 cell line phenotypically distinct. In Fig. 3I we can see the relation between CD326 and CD44 and the distribution of the MIA PaCa-2 viable cell population according to four possible phenotypes: Q1 (blue), CD44^−^CD326^+^, comprising 6.9% of total cells; Q2 (purple), CD44^+^CD326^+^, 35.5% of cells; Q3 (brown), CD44^−^CD326^−^, 9.6% of cells; and Q4 (green), CD44^+^CD326^−^, 48% of cells. Thus, in the MIA PaCa-2 cell line, there is an overwhelming predominance of the phenotypes CD44^+^CD326^−^ (48%) and CD44^+^CD326^+^ (35.5%).

**Figure 4:**
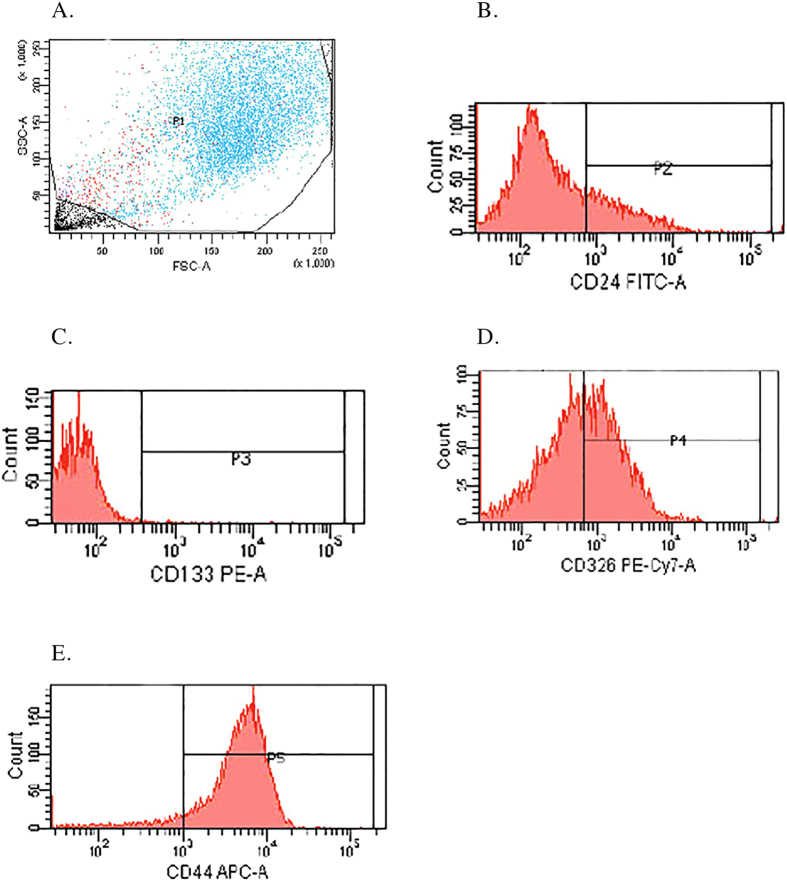
Flow cytometry analysis of morphology and stem cell marker expression on PANC-1 tumor cells. (**A**) Dot plot FSC/SSC representative of the population P1 (blue) population; (**B**) Histogram representative of CD24 expression in P1; (**C**) Histogram representative of CD133/1 expression in P1; (**D**) Histogram representative of CD326 expression in P1; (**E**) Histogram representative of CD44 expression in P1.

When performing the same study in the PANC-1 cell line (Fig. 4), while in optic microscopy and immunohistochemistry we observed three distinct populations, flow cytometry dot plot FSC/SSC revealed the existence of only one population of viable cells (P1). In fact, in 10,000 events, a population (P1) with 6,888 events was obtained (Fig. 4A), corresponding to 68.9% of total cells, with an MFI of 1,192, supporting the possibility of the existence of a pleomorphic cell line. To investigate the presence of stem cells using CD24 FITC, CD44 APC, CD133/1 PE and CD326 PE-Cy7, we have characterized this population (P1) in relation to each marker and performed the gates P2, P3, P4 and P5. P2 corresponds to a subpopulation expressing CD24 (Fig. 4B), comprising 23.4% of P1, with an MFI of 3,502; P3 corresponds to a subpopulation expressing CD133/1 (Fig. 4C), 0.7% of P1, with an MFI of 3,324; P4 corresponds to a subpopulation expressing CD326 (Fig. 4D), 49.1% of P1, with an MIF of 2,068; and P5 corresponds to a subpopulation expressing CD44 (Fig. 4E), 92.3% of P1, with an MFI of 1,186. PANC-1 cells seem to have the phenotype CD24^−/+^, CD44^+^, CD326^−^/^+^ and CD133/1^−^.

#### Genetic profile

**Figure 5:**
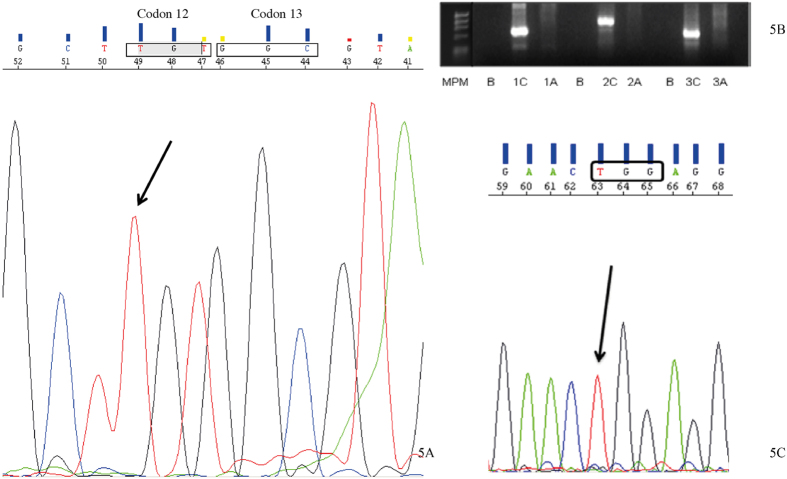
MIA PaCa-2 genetic profile. (**A**) Codon 12 of *KRAS*. The arrow signals a homozygous missense mutation (p.G12C; GGT > TGT); (**B**) Electrophoresis results from the amplification products of exons 1, 2 and 3 of the gene *CDKN2A/p16*^*INK4A*^. A - cell line samples; B - contamination control, without DNA (blanks); C - normal samples (positive controls); 1, 2, 3 – exons; MPM - Molecular weight marker φX174 DNA-HaeIII; (**C**) Exon 7 of *TP53*. The arrow signals a homozygous missense mutation (p.R248W; CGG > TGG).

The genotyping of MIA PaCa-2 (Fig. 5) confirmed the presence of a homozygous missense mutation in codon 12 (p.G12C; GGT > TGT) of *KRAS* (Fig. 5A), a homozygous deletion encompassing exons 1, 2 and 3 of the *CDKN2A/p16*^*INK4A*^ gene (Fig. 5B) and a homozygous missense mutation in exon 7 (p.R248W; CGG > TGG) of *TP53* (Fig. 5C). Sequencing analysis did not identify any mutation in SMAD4/DPC4, nor was any microsatellite instability found.

**Figure 6:**
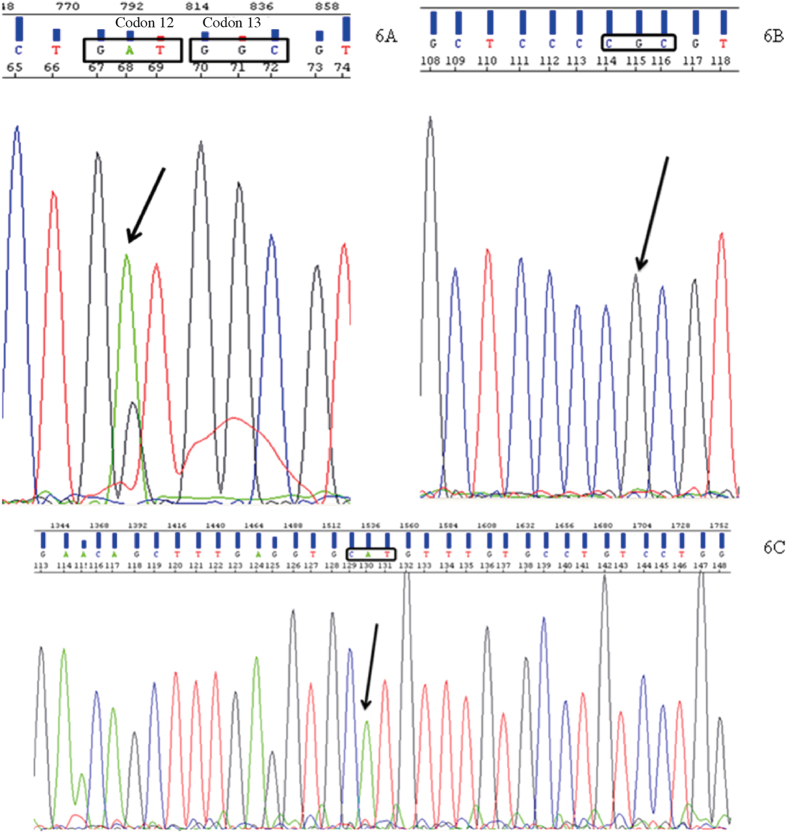
PANC-1 genetic profile. (**A**) Codon 12 of *KRAS*. The arrow signals a heterozygous missense mutation (p.G12D; GGT > GAT); (**B**) Exon 4 of *TP53*. The arrow signals a homozygous missense mutation (p.P72R; CCC > CGC); (**C**) Exon 8 of *TP53*. The arrow signals a homozygous missense mutation (p.R273H; CGT > CAT).

The genotyping of PANC-1 (Fig. 6) revealed a heterozygous missense mutation in codon 12 (p.G12D; GGT > GAT) of *KRAS* (Fig. 6A), a homozygous deletion of exons 1, 2 and 3 of the *CDKN2A/p16*^*INK4A*^ gene (Fig. 5B) and two missense variants in the *TP53* gene, one in exon 4 (p.P72R; CCC > CGC) (Fig. 6B) and another in exon 8 (p.R273H; CGT > CAT) (Fig. 6C). As with the MIA PaCa-2 cell line, no SMAD4/DPC4 mutation was found by sequencing and no microsatellite instability was highlighted.

## Discussion

Microscopic observation of MIA PaCa-2 cells revealed (Fig. 1) the presence of two distinct morphological patterns: large cells and small cells (morula), while such observation of PANC-1 cells revealed (Fig. 2) the presence of three distinct morphological patterns: large cells (isolated), intermediate cells (stellate) and small cells (morula). Flow cytometry was used to clarify whether in morphological terms there were in fact two cell populations in MIA Paca-2 and three in PANC-1. MIA PaCa-2 flow cytometry results (Fig. 3) demonstrated the presence of two groups of viable cells: the population of large cells (blue) and that of small cells (green). This result may support the possibility that a polymorphic cell line exists. PANC-1 flow cytometry results demonstrated the presence of only one population of viable cells, supporting the possibility that a pleomorphic cell line exists. However, since there are no specific markers of pancreatic tumor cells, it cannot be stated with absolute certainty that this is a case of polymorphism for cell line MIA PaCa-2 and pleomorphism for cell line PANC-1.

The MIA PaCa-2 cell line (Table 1 and Fig. 1) expresses CK5.6, AE1/AE3, E-cadherin, vimentin, chromogranin A, synaptophysin, SSTR2 and NTR1, but not CD56. These cells are epithelial as they express CK5.6, AE1/AE3 and E-cadherin, with mesenchymal characteristics because they express vimentin, neuroendocrine differentiation since they express chromogranin A and synaptophysin, and hormonal receptors because they express SSTR2 and NTR1. The presence of SSTR2 and NTR1 may enable peptide receptor radionuclide therapy to be used in this cell line.

PANC-1 cells (Table 2 and Fig. 2) do not express E-cadherin, synaptophysin or NTR1. They are cells with epithelial characteristics since they express CK5.6 and AE1/AE3, mesenchymal characteristics because they express vimentin, neuroendocrine differentiation as they express chromogranin A and CD56, and hormonal receptors because they express SSTR2. The presence of SSTR2 may allow peptide receptor radionuclide therapy to be used in this cell line.

If we compare the immunohistochemical pattern of these two cell lines (Tables 1 and 2), we notice that it is different, not only due to the absence of expression of certain markers and receptors, but also because of the variable expression of others. Thus, in the large cells of MIA PaCa-2 there is a more pronounced expression of CK5.6, AE1/AE3, E-cadherin, vimentin, synaptophysin, SSTR2 and NTR1. The large cells of PANC-1, on the other hand, have a more pronounced expression of chromogranin A and CD56. In the small cells of MIA PaCa-2 there is a more pronounced expression of CK5.6 and AE1/AE3, and to an even greater extent E-cadherin, chromogranin A and NTR1. In the small cells of PANC-1 there is a more pronounced expression of vimentin. This difference in cell marker and receptor expression between large and small cells in the same cell line, and between large and small cells in both cell lines, enhances the concept of population variability.

Concerning NED, MIA PaCa-2 expressed chromogranin A and synaptophysin (Table 1), and PANC-1 expressed chromogranin A and CD56 (Table 2). In pancreatic carcinoma NED has been closely associated with tumor behavior: patients with tumors harboring NED have a better overall survival and NED seems to be an independent predictor of survival after surgery^7^. Both cell lines expressed chromogranin A, and this observation reveals NED to be associated with a better prognosis^32^,^33^.

Both MIA PaCa-2 and PANC-1 cells expressed epithelial markers CK5.6 and AE1/AE3, and the mesenchymal marker vimentin, allowing them to be characterized as epithelial-mesenchymal cells. The activation of an EMT program is the essential mechanism for the acquisition of a malignant phenotype by epithelial cancer cells, and the hallmark of EMT is the loss of the epithelial homotypic adhesion molecule E-cadherin and gain of mesenchymal marker vimentin^34^. PANC-1 had no E-cadherin expression. It has been demonstrated that EMT contributes to drug resistance in pancreatic cancer and that increased expression of E-cadherin is associated with improved survival in several tumor types^35^. In some reports of migration assays, it was demonstrated that migration of PANC-1 cells was greater than MIA PaCa-2 cells on transwell^®^ plates coated with collagen type I^36^. Although not conclusive, the invasive behavior through matrigel^®^ was also demonstrated for MIA PaCa-2 and PANC-1^36^. Our results indicate that MIA PaCa-2 and PANC-1 have EMT potential but that of PANC -1 is superior.

Concerning stem cell marker expression, both cell lines expressed population heterogeneity, with MIA PaCa-2 (Fig. 3) cells expressing CD24^−^CD44^+/++^CD326^−/+^CD133/1^−^. It seems that the large cell population has the phenotype CD24^−^CD44^++^CD326^−^CD133/1^−^ and the small cell population, CD24^−^CD44^+^CD326^−/+^CD133/1^−^. We also found that MIA PaCa-2 had two relevant CD44^+^ CD326^−^ (48%) and CD44^+^CD326^+^ (35.5%) populations (Fig. 3I). The PANC-1 cell line expressed the phenotype CD24^−/+^CD44^+^CD326^−/+^CD133/1^−^ (Fig. 4). CD24^+^CD44^+^CD326^+^ cancer cells display the ability to self-renew, generate different progeny and recapitulate the phenotype of the tumor from which they were derived^37^. Cells negative for all three markers are not able to initiate pancreatic cancer until 10^4^ or more cells are implanted^38^. Li *et al.*^38^ demonstrated that cells expressing CD24^+^CD44^+^CD326^+^ formed 6 tumors in 12 (50%) NOD/SCID mice after they were injected with 100 cells subcutaneously in their flank. With the single marker CD44^+^ or with the dual markers CD44^+^CD326^+^, 4 out of 16 (25%) animals developed tumors when injected with as few as 100 cells, and when the number of cells injected was increased (500 > 10^3^ > 10^4^), the dual marker combination CD44^+^CD326^+^ resulted in an enhanced tumorigenic potential compared with the single marker CD44^+^. CD24^+^ cells are less tumorigenic than CD44^+^ cells, with 1 out of 16 (6.25%) animals developing tumors when injected with as few as 100 cells. With CD326, 8 out of 18 (44.4%) and 1 out of 18 (5.6%) animals developed tumors when injected with 500 CD326^+^ and 500 CD326^−^ cells respectively. 10^3^ CD24^−^ cells were needed to generate a tumor when injected in 16 animals. We may presume that PANC-1 cells CD24^+^CD44^+^CD326^+^ are more tumorigenic than MIA PaCa-2 cells CD24^−^CD44^+^CD326^+^, and that within MIA PaCa-2 the subpopulation CD44^++^CD326^+^ is more tumorigenic than the subpopulation CD44^+^CD326^−^. The surface marker CD133 was first reported to have the properties of CSCs in brain and colon cancer^39^. Results from Hermann *et al.*^40^ revealed that 500 CD133^+^ cells injected in NOD/SCID mice were able to generate tumors. Lee *et al.*^41^ demonstrated that CD133^+^ cells had a higher tumorigenic potential and higher rates of metastasis to the lung than CD44^+^ cells, suggesting that CD133 is implicated in the aggressive behavior of pancreatic cancer. Neither MIA PaCa-2 nor PANC-1 expressed CD133, which seems to reduce their potential to metastasize. Despite these results, we should point out that the presence of stem cells cannot be inferred purely from surface marker expression. It is also known, for example, that the expression of the markers CD24, CD44 and CD326 varies with the local microenvironment or niche^42^. Functional analyses should therefore be performed, such as sphere formation assays^43^ or tumorigenesis in a transplantation setting in immunocompromised mice^44^,^45^.

*KRAS*, *CDKN2A/p16*^*INK4A*^, *TP53*, *SMAD4/DPC4* and MSI analyses were used to throw light on the genetic carcinogenic mechanisms involved and identify possible sources of resistance to chemo and radiotherapy. Genotyping of MIA PaCa-2 (Fig. 5A) and PANC-1 (Fig. 6A) confirmed the presence of a homozygous (p.G12C; GGT > TGT) and a heterozygous missense mutation (p.G12D; GGT > GAT) in codon 12 of *KRAS* respectively. This is a hotspot codon well known for interfering with the KRAS GTPase function, keeping the molecule in its GTP-bound state, thus allowing uninterrupted activation of downstream effector pathways such as mitogen-activated protein kinase (MAPK)^46^. *KRAS* mutations confer drug resistance and lead to aggressive tumor growth and metastasis and a poor clinical outcome^47^. Moreover, tumor cells with a mutant *KRAS* are more radiation-resistant than are cells with the wild type of *KRAS*^48^. Some experiments have shown that farnesyltransferase inhibitor drugs, which prevent the post-translation processing of *KRAS*, essential for its appropriate cell-membrane localization, sensitize *KRAS*-mutated pancreatic cancer cells to radiation^49^.

*CDKN2A*, located on chromosome 9p21, encodes two proteins, P16 (INK4) and P14 (ARF). Both gene products have an independent first exon (exon 1-α and exon 1-β, respectively) but share exons 2 and 3, and are translated in different reading frames^50^. These proteins are involved in the negative control of cell proliferation: P16 (INK4) is a cyclin-dependent kinase inhibitor that induces G1 cell-cycle arrest by inhibiting Cdk4/6 and hence keeping the retinoblastoma protein (RB) in its active, dephosphorylated state; P14 (ARF) acts both at G1/S and G2/M phases in a TP53-dependent manner by destabilizing MDM2, the protein that maintains TP53 at low levels^51^. Concerning pancreatic tumors, the *CDKN2A/p16*^*INK4A*^ gene is inactivated in 95% of invasive carcinomas, 40% by homozygous deletion, 40% by an intragenic mutation coupled with loss of the second allele and 10–15% of cases by gene promoter hypermethylation. Tumors with *CDKN2A* deletion are larger and patients may have a survival period which is significantly shorter^52^. The genotyping of MIA PaCa (Fig. 5B) and PANC-1 (Fig. 5B) confirmed the presence of a homozygous deletion encompassing exons 1, 2 and 3 of the *CDKN2A/p16*^*INK4A*^ gene, resulting in the indirect impairment of two important tumor suppressor proteins, RB and TP53, thus reproducing the effect of inactivating mutations.

*TP53* function also seems to be disrupted in the two cell lines by missense mutations. The MIA PaCa-2 cell line showed a pathogenic missense variant in a hotspot codon (p.R248W). The PANC-1 cell line had a common polymorphism in exon 4 (p.P72R; rs1042522), located in the proline-rich domain of *TP53* and possibly associated with differences in apoptosis induction^53^, and a pathogenic missense variant in an exon 8 hotspot codon (p.R273H; rs28934576). The two pathogenic variants, in exons 7 and 8, correspond to the DNA binding domain of TP53 and are known to disrupt its function as a transcription activator^54^,^55^. There is also some evidence that some *TP53* mutations, including R273H, may induce gain of function effects, as knock-in mice expressing this mutation showed increased metastases compared with *TP53* knock-out mice^56^ and a different cancer distribution. *TP53* mutations were found in homozygosity in both cell lines, most probably due to the loss of heterozygosity (LOH) associated with chromosome 17p deletions including the wild-type allele, correlating with the loss of *TP53* suppressor function.

No changes were found in MIA PaCa-2 or PANC-1 concerning *SMAD4/DPC4.* Furthermore, the absence of MSI from all of the five markers analyzed suggests that the DNA mismatch repair (MMR) system is preserved^57^,^58^ in the MIA PaCa-2 and PANC-1 cell lines.

In conclusion, MIA PaCa-2 expresses polymorphism and PANC- 1 expresses pleomorphism. Polymophism and pleomorphism had not yet been described in previous studies, and they may introduce variations in the distribution of stem cell markers, making tumors more resistant to therapy. However, the association between stem cell markers and a specific type of cell morphology was not proved in this study. If this association exists, the treatment of these tumors may be even more complex than we currently assume, due to their heterogeneity.

It was confirmed that both cell lines have EMT differentiation but since PANC-1 has no E-cadherin, its behavior is more aggressive with a greater metastasizing potential.

Both cell lines express neuroendocrine differentiation and SSTR2, and this had not been established in previous studies. It is precisely these features which make them suitable for pancreatic cancer neuroendocrine chemotherapy and peptide receptor radionuclide therapy.

## Materials and Methods

### Cell culture conditions

The MIA PaCa-2 (CRL-1420) and PANC-1 (CRL-1469) cell lines were both purchased from American Tissue Cell Culture (ATCC, Manassas, VA, USA), and maintained under culture with Dulbecco’s Modified Eagle’s Medium (DMEM) (ATCC 30-2002, Manassas, VA, USA) supplemented with heat-inactivated fetal bovine serum (FBS) (ATCC 30-2020) to a final concentration of 10% (v/v), at 37 °C and in a 5% CO_2_ atmosphere. In the case of MIA PaCa-2, horse serum (HS) (ATCC 30-2040, Manassas, VA, USA) to a final concentration of 2.5% was also added. Every 2–3 days cells were passaged (subcultivation ratio 1:3) using trypsinization for cells to detach in order to maintain them in culture. A passage number of 20 was assumed for each cell line; after these 20 passages a new culture was started with the stock constituted after the reception of the cells from ATCC. This stock consisted of about 30 vials obtained during the first five passages; the vials were subsequently stored in liquid nitrogen, with complete growth medium 95% and DMSO 5%.

### Immunohistochemistry

The immunohistochemical analysis was performed on sterile 10cm^2^ Lab-Tek Flaskette glass slides (VWR International, Ref. NUNC 177453) previously seeded with MIA PaCa-2 and PANC-1 cells. As immunohistochemical markers, we selected epithelial (CK5/6, AE1/AE3, E-cadherin), mesenchymal (vimentin), endocrine (chromogranin A, CD56, synaptophysin) and hormonal (SSTR2, NTR1) antibodies. CK5.6, CD56 and synaptophysin were applied to the automated slide staining system BenchMark Ultra, with ultraView Universal DAB Detection kit 760-500, both from Ventana. The immunohistochemical staining of AE1/AE3, E-cadherin, vimentin, chromogranin A, SSTR2 and NTR1 was performed manually, the first four with the Ultravision Polyvalent HRP (TP-125-HL) kit from Lab Vision, and the last two with Bond™ Polymer Refine Detection from Leica Biosystems.

Primary antibodies against CK5.6 (clone D5/16B4; Cell Marque, California, USA), at a dilution of 1:50 for 40 minutes, with skin cells as positive control, were applied to the sections and incubated at room temperature, as were ready-to-use AE1/AE3 (clone AE1/AE3; Novocastra Laboratories, Ltd., Newcastle, UK), for 30 minutes, with appendix cells as positive control; E-cadherin (clone 36B5; Novocastra Laboratories Ltd., Newcastle, UK), at a dilution of 1:100 for 30 minutes, with tonsil cells as positive control; vimentin (clone VIM 3B4; DakoCytomation, Glostrup, Denmark), at a dilution of 1:200 for 30 minutes, with colon cells as positive control; chromogranin A (clone DAK-A3; DakoCytomation, Glostrup, Denmark), at a dilution of 1:300 for 30 minutes, with pancreas cells as positive control; CD56 (clone 123C3; DakoCytomation, Glostrup, Denmark), at a dilution of 1:100 for 44 minutes, with colon cells as positive control; synaptophysin (polyclonal; Thermo Scientific, MA, USA), at a dilution of 1:50 for 36 minutes, with thyroid cell as positive control; SSTR2 (polyclonal; abcam (ab9550), Cambridge, United Kingdom), at a dilution of 1:300 for 30 minutes, with kidney cells as positive control; and NTR1 (polyclonal H-130; Santa Cruz Biotechnology (sc-15311), California, United States), at a dilution of 1:250 for 30 minutes, with a pancreatic xenographt as positive control. They were washed with phosphate-buffered saline (PBS) (TP-125-PB; Lab Vision Corporation; Fremont CA; USA), after which slides were incubated with biotin-labeled secondary antibody, biotynilated goat anti-polyvalent (TP-125-BN; Lab Vision Corporation; Fremont CA; USA), for 15 minutes. Primary antibody binding was localized in tissues using peroxidase-conjugated streptavidin (TP-125-HR; Lab Vision Corporation; Fremont CA; USA), and 3,3′-diaminobenzidine tetrahydrochloride (DAB) (RE7190-K; Novocastra Laboratories Ltd., Newcastle, UK) was used as chromogen, according to the manufacturer’s instructions. Hematoxylin was used to counterstain the slides, which were then dehydrated and mounted. As negative controls, we used positive controls without the addition of a primary antibody.

The intensity of the staining was graded semi-quantitatively on a three point scale [+ (low), ++ (medium), +++ (high)] and the percentage of immunostained cells was also registered. The final score was obtained by establishing a scale, having adopted the following threshold of reactivity (*cut-off*  ) : −(0%); + (<10%), ++ (10–75%), +++ (>75%).

### Flow Cytometry

Flow cytometry was used for identification and clarification of the cell population for each cell line. MIA Paca-2 and PANC-1 cell lines were dissociated using 0.25% trypsin/0.53 mM EDTA in Hanks Balanced Salt Solution (ATCC 30-2101) for 5 minutes at 37 °C. Trypsin was then inhibited with DMEM complemented with a 10% FBS culture medium (MIA PaCa-2 and PANC-1) and 2.5% HS (MIA PaCa-2). Approximately 0.5 × 10^6^ cells were transferred to a 5 ml tube (BD Biosciences, San Jose, CA, USA) and washed twice with Dulbecco’s Phosphate Buffered Saline (PBS). Cells were resuspended in 500 μl PBS, and samples were acquired on a FACSCanto II flow cytometer (BD Biosciences) and analyzed with FACSDiva software (BD Biosciences). Thus, it was possible to obtain, select and represent the cells graphically in the form of a *dot plot* as a function of forward-scattered (*FSC*) and side-scattered light (*SSC*). We used the 7-AAD (7-amino-actinomycin-D) as a dead cell marker to confirm the presence of the non-viable population, whereby the livings cells were quantified by exclusion.

Flow cytometry was also used for the identification of pancreatic cancer *stem* cells markers^10^, such as CD24, CD44, CD133/1 and CD326. After dissociation with trypsin and subsequent neutralization with DMEM complemented with FBS and HS, cells were resuspended in 500 μl PBS and, for MIA PaCa-2, 20 μl of the following monoclonal antibodies were added: anti-CD24-PE (clone 32D12, Miltenyi Biotec), anti-CD44-PerCP/Cy5.5 (clone IM7, BD Pharmingen), anti-CD133/1-APC (clone AC133, Miltenyi Biotec) and anti-CD326-PE-Cy7 (clone 9C4, Miltenyi Biotec). For PANC-1, 20 μl of the following monoclonal antibodies were added: anti-CD24-FITC (clone ML5, BD Pharmingen), anti-CD44-APC (clone BJ18, Biolegend), anti-CD133/1-PE (clone AC133, Miltenyi Biotec) and anti-CD326-PE-Cy7 (clone 9C4, Biolegend). The mixture was incubated for 20 minutes at room temperature and protected from light. After incubation, cells were washed twice with PBS and resuspended in 500 μl PBS. For MIA PaCa-2, rat anti-mouse IgG1-PE, IgG2b-PerCP/Cy5.5, IgG1-APC and IgG2b-PE-Cy7 were used as isotype negative controls (Biolegend) for CD24, CD44, CD133/1 and CD326 respectively. For PANC-1, rat anti-mouse IgG2a-FITC, IgG1-APC, IgG1-PE, and IgG2b-PE-Cy7 were used as isotype negative controls (Biolegend) for CD24, CD44, CD133/1 and CD326 respectively. Samples were acquired on a FACSCanto II flow cytometer (BD Biosciences) and analyzed with FACSDiva software (BD Biosciences).

### PCR and sequencing

By determining the genetic profile of cell lines MIA PaCa-2 and PANC-1, this study aimed to investigate the most frequent mutations in pancreatic ductal adenocarcinoma. DNA was extracted from 2 to 5 × 10^6^ viable cells of the MIA PaCa-2 and PANC-1 cell lines using the QIAamp DNA mini Kit (Qiagen, Hilden, Germany), and from the blood of healthy control individuals using the QIAamp DNA blood mini Kit (Qiagen, Hilden, Germany), according to the manufacturer’s protocol.

*KRAS* (codons 12, 13 and 61), *TP53* (exons 4 to 8) and *SMAD4/DPC4* (codifying exons 2 to 11) were studied by sequencing using primers and cycling conditions, as described in Table 3. Sequencing was performed using the ABI PRISM BigDye v1.1 Terminator Cycle Sequencing Kit (Applied Biosystems, Foster City, CA, USA), the same primers as in PCR, and an ABI PRISM 3130 Genetic Analyzer (Applied Biosystems, Foster City, CA, USA). Results were analyzed with sequencing analysis software v 5.2 (Applied Biosystems, Foster City, CA, USA). For *CDKN2A/p16*^*INK4A*^, as the most frequent mutation is the homozygous deletion, we only proceeded to PCR and thus three exons, exons 1-α, 2 and 3, were amplified from the DNA of cell lines and of a healthy control using primers and the conditions described in Table 3. MSI testing was carried out with a multiplex PCR amplification of 5 *quasi* monomorphic mononucleotide repeats (BAT25, BAT26, NR21, NR22 and NR24) with DNA from the cell lines and from the healthy control. Briefly, 40 ng of genomic DNA were amplified in a 25 μl reaction using primers at 0.16 μM, MgCl_2_ at 1.5 mM, dNTPs at 200 μM and 0.2 U recombinant Taq DNA polymerase (Fermentas, Thermo Scientific, MA, USA). Primer sequences and PCR cycling conditions are described in Table 4. A volume varying from 0.1 to 1 μl of amplified PCR product was added to 25 μl of formamide and 1 μl of internal fluorescence standard-sized GS500LIZ, incubated for 5 minutes at 95 °C and then applied to the ABI PRISM 3130 Genetic Analyzer using the POP7 polymer. Automatic fragment analysis was carried out with GeneMapper software v3.7 (Applied Biosystems, Foster City, CA, USA). The presence of three or more mutant alleles was considered to indicate the presence of MSI-high (MSI-H)^59^. The absence of MSI implies the absence of mutations in the MMR system.

## Additional Information

**How to cite this article**: Gradiz, R. *et al.* MIA PaCa-2 and PANC-1 – pancreas ductal adenocarcinoma cell lines with neuroendocrine differentiation and somatostatin receptors. *Sci. Rep.* **6**, 21648; doi: 10.1038/srep21648 (2016).

## Figures and Tables

**Table 1:** MIA PaCa-2 immunohistochemical profile.

Marker	Morphology	Result	Morphology	Result
CK 5.6	Large cells	+++	Small cells (morula)	++
AE1/AE3	Large cells	+++	Small cells (morula)	+++
E-cadherin	Large cells	+++	Small cells (morula)	+++
Vimentin	Large cells	+++	Small cells (morula)	++
Chromogranin A	Large cells	−/+	Small cells (morula)	+++
CD 56	Large cells	−/+	Small cells (morula)	−
Synaptophysin	Large cells	+	Small cells (morula)	−
SSTR2	Large cells	+++	Small cells (morula)	+++
NTR1	Large cells	++	Small cells (morula)	++

Cut off: −(0%); + (<10%); ++ (10–75%); +++ (>75%).

**Table 2:** PANC-1 immunohistochemical profile.

Marker	Morphology	Result	Morphology	Result	Morphology	Result
CK 5.6	Large cells	++	Intermediate cells (stellate)	++	Small cells (morula)	−
AE1/AE3	Large cells	++	Intermediate cells (stellate)	++	Small cells (morula)	+++
E-cadherin	Large cells	−	Intermediate cells (stellate)	−	Small cells (morula)	−
Vimentin	Large cells	++	Intermediate cells (stellate)	++	Small cells (morula)	+++
Chromogranin A	Large cells	+	Intermediate cells (stellate)	+	Small cells (morula)	−
CD 56	Large cells	+	Intermediate cells (stellate)	+	Small cells (morula)	−
Synaptophysin	Large cells	−	Intermediate cells (stellate)	−	Small cells (morula)	−
SSTR2	Large cells	+*	Intermediate cells (stellate)	+	Small cells (morula)	++
NTR1	Large cells	−	Intermediate cells (stellate)	−	Small cells (morula)	−

Cut off: −(0%); + (<10%); ++ (10–75%); +++ (>75%); *polarized cytoplasm.

**Table 3:** Sequences of *primers* and *annealing* temperatures used in *KRAS*, *CDKN2A/p16*^*INK4A*^, *TP53* and *SMAD4/DPC4* amplification.

Genes	Codões	Primers	Annealing temperatures
*KRAS*	12 e 13	F: 5′-ACATGTTCTAATATAGTCAC-3′	54 °C
		R: 5′-CTATTGTTGGATCATATTCG-3′	
	61	F: 5′-TTCCTACAGGAAGCAAGTAGT-3′	56 °C
		R: 5′-CATGGCATTAGCAAAGACTC-3′	
*CDKN2A/ p16*^*INK4A*^	Exon 1-alpha	F: 5′-ACC GGA GGA AGA AAG AGG AG-3′	58 °C
		R: 5′-TCA GGT AGC GCT TCG ATT CT-3′	
	Exon 2	F: 5′-GTG AGG GGG CTC TAC ACA AG-3′	60 °C
		R: 5′-CAG CAC AGA AAG TTC AGC CC-3′	
	Exon 3	F: 5′-TAC ATG CAC GTG AAG CCA TT-3′	56 °C
		R: 5′-TTC CCC CAC TAC CGT AAA TG-3′	
*TP53*	Exon 4	F: 5′-TGA CTG CTC TTT TCA CCC A T-3′	59 °C
		R: 5′-GGA AGC CAG CCC CTC AGG GC-3′	
	Exon 5	F: 5′-AAC TCT GTC TCC TTC CTC TT-3′	58 °C
		R: 5′-GCC CCA GCT GCT CAC CAT CGC TA-3′	
	Exon 6	F: 5′- TCT GAT TCC TCA CTG ATT GC-3′	54 °C
		R: 5′-CCA GAG ACC CCA GTT GCA AA-3′	
	Exon 7	F: 5′- GCT GAG GAA GGA GAA TGG-3′	57 °C
		R: 5′-GTG ATG AGA GGT GGA TGG-3′	
	Exon 8	F: 5′-CCT CTT AAC CTG TGG CTT CTC-3′	58 °C
		R: 5′-TAA CTG CAC CCT TGG TCT CCT-3′	
*SMAD4/DPC4*	Exon 1	F: 5′-CGTTAGCTGTTGTTTTTCACTG-3′	54 °C
		R: 5′-ACAGTATCTGAAGAGATGGAG-3′	
	Exon 2	F: 5′-TGTATGACATGGCCAAGTTAG-3′	51 °C
		R: 5′-CAATACTCGGTTTTAGCAGTC-3′	
	Exon 3	F: 5′-CTGAATTGAAATGGTTCATGAAC-3′	51 °C
		R: 5′-GCCCCTAACCTCAAAATCTAC-3′	
	Exon 4	F: 5′-TTTTGCTGGTAAAGTAGTAGC	50 °C
		R: 5′-CTATGAAAGATAGTACAGTTAC	
	Exões 5 e 6	F: 5′-CATCTTTATAGTTGTGCATTATC-3′	52 °C
		R: 5′-TAATGAAACAAAATCACAGGATG-3′	
	Exon 7	F: 5′-TGAAAGTTTTAGCATTAGACAAC-3′	50 °C
		R: 5′-TGTACTCATCTGAGAAGTGAC-3′	
	Exon 8	F: 5′-TGTTTTGGGTGCATTACATTTC-3′	52 °C
		R: 5′-CAATTTTTTAAAGTAACTATCTGA-3′	
	Exon 9	F: 5′ – TATTAAGCATGCTATACAATCTG-3′	50 °C
		R: 5′-CTTCCACCCAGATTTCAATTC-3′	
	Exon 10	F: 5′-AGGCATTGGTTTTTAATGTATG-3′	52 °C
		R: 5′-CTGCTCAAAGAAACTAATCAAC-3′	
	Exon 11	F: 5′-CCAAAAGTGTGCAGCTTGTTG-3′	54 °C
		R: 5′-CAGTTTCTGTCTGCTAGGAG-3′	

F – Forward; R – Reverse. The same *primers* were used for the first PCR and for sequencing. The *annealing* temperatures correspond to those of the first PCR.

**Table 4:** Sequences of *primers* used in the detection of microsatellite instability.

Marker	GenBank Ref	Repetitions	Primer	Fluorochrome	Product (bp)
		Location			
BAT25	U41210	26A	F^1^: 5′-TCGCCTCCAAGAATGTAAGT-3′	6-FAM	110–130
		*CKIT*; intron 5	R: 5′-TCTGCATTTTAACTATGGCTC-3′		
BAT26	L04143	25T	F^2^: 5′-TGACTACTTTTGACTTCAGCC-3′	TET/VIC	100–120
		*MSH2*: intron 16	R: 5′-AACCATTCAACATTTTTAACCC-3′		
NR21	XM033393	21(T)	F^3^: 5′-TAAATGTATGTCTCCCCTGG-3′	HEX/NED	103
		*SLC7A8*; 5′ UTR	R: 5′-ATTCCTACTCCGCATTCACA-3′		
NR22	L38961	22(T)	F^1^: 5′-GAGGCTTGTCAAGGACATAA-3′	FAM	142
		*ITM1*; 3′ UTR	R: 5′-AATTCGGATGCCATCCAGTT-3′		
NR24	X60152	24(T)	F^3^: 5′-CCATTGCTGAATTTTACCTC-3′	HEX/NED	132
		*ZNF-2*; 3′ UTR	R: 5′-ATTGTGCCATTGCATTCCAA-3′		

F – Forward; R – Reverse; bp – base pairs of the amplified product. The microsatellites were amplified with a multiplex PCR and with an *annealing* temperature of 55 °C.
